# ASSOCIATION OF INTERLEUKIN-10 -592A>C AND -819T>C POLYMORPHISMS WITH GASTRIC CANCER RISK: A SYSTEMATIC REVIEW AND META-ANALYSIS OF 44 CASE-CONTROL STUDIES

**DOI:** 10.1590/0102-672020180001e1415

**Published:** 2019-01-07

**Authors:** Mansour MOGHMI, Amir ARJMANDI, Kazem AGHILI, Mohammadali JAFARI, Masoud ZARE-SHEHNEH, Shohreh RASTEGAR, Seyed Mojtaba ABOLBAGHAEI, Hossein NEAMATZADEH

**Affiliations:** 1 Shahid Sadoughi University of Medical Sciences, Pathology, Yazd, Yazd;; 2 Shahid Sadoughi University of Medical Sciences, Medical Genetics, Yazd, Yazd;; 3 Shahid Sadoughi University of Medical Sciences, Radiology, Yazd, Yazd;; 4 Shahid Sadoughi University of Medical Sciences, Emergency Medicine, Yazd, Yazd;; 5 Shahid Sadoughi University of Medical Sciences, Anesthesiology, Yazd, Yazd;; 6 Shahid Beheshti University of Medical Sciences, Forensic Medicine, Tehran, Tehran, Iran.

**Keywords:** Stomach neoplasms, Interleukin-10, Polymorphism, genetic, Meta-analysis, Neoplasias Gástricas, Interleucina-10, Polimorfismo genético, Metanálise

## Abstract

**Introduction:**

A series of studies have evaluated the association between -592A>C and -819T>C polymorphisms in the promoter regions of Interleukin-10 (IL-10) and gastric cancer (GC) risk. However, the results remain inconclusive.

**Objective:**

To better understand the association of the polymorphisms with GC risk, we performed a comprehensive meta-analysis.

**Method:**

An electronic search was performed of several databases to identify relevant studies up to April 2018.

**Results:**

A total of 44 case-control studies, including 26 studies on IL-10 -592A>C (5,332 cases and 8,272 controls) and 18 studies on IL-10 -819T>C (3,431 cases and 6,109 controls) were selected. Overall, -592A>C polymorphism was associated with the risk of GC under the heterozygote model (OR=1.153, 95% CI=1.020-1.305, p=0.023), but not -819T>C polymorphism. When stratified by ethnicity, significant association was only observed in the Asians under the allele model (OR=1.153, 95% CI=1.007-1.320, p=0.040) and the heterozygote model (OR=1.218, 95% CI=1.076-1.379, p=0.002) for -592A>C.

**Conclusion:**

The current meta-analysis results inconsistent with previous meta-analyses; showed that the IL-10 -592A>C polymorphism, but not -819T>C polymorphism, may be contributed to the susceptibility of GC in overall and Asian populations.

## RESUMO

**Introdução:**

Uma série de estudos avaliou a associação entre os polimorfismos -592A>C e -819T>C nas regiões promotoras do risco de interleucina-10 (IL-10) e câncer gástrico (GC). No entanto, os resultados permanecem inconclusivos.

**Objetivo:**

Para entender melhor a associação dos polimorfismos com o risco de GC, realizamos uma meta-análise abrangente.

**Método:**

Foi realizada busca eletrônica de vários bancos de dados para identificar estudos relevantes até abril de 2018.

**Resultados:**

Um total de 44 estudos caso-controle, incluindo 26 estudos sobre IL-10 -592A>C (5.332 casos e 8.272 controles) e 18 estudos sobre IL-10 -819T>C (3.431 casos e 6.109 controles) foram selecionados. No geral, o polimorfismo -592A> C foi associado ao risco de GC sob o modelo heterozigoto (OR=1,153, 95% IC=1,020-1,305, p=0,023), mas não polimorfismo -819T>C. Quando estratificada por etnia, associação significativa foi observada apenas nos asiáticos sob o modelo alelo (OR=1,153, IC 95%=1,007-1,320, p=0,040) e o modelo heterozigoto (OR=1,218, IC 95%=1,076-1,379, p=0,002) para -592A>C.

**Conclusão:**

Os atuais resultados são inconsistentes com metanálises anteriores; mostrou que o polimorfismo IL-10 -592A> C, mas não o polimorfismo -819T>C, pode ter contribuído para a suscetibilidade de GC em populações globais e asiáticas.

## INTRODUCTION

Gastric cancer (GC) is the 5^th^ most common cancer and second leading cause of cancer-related deaths globally ^21^ ^,^ ^32^ ^,^ ^36^. In 2016 there were an estimated 26,370 new cases of GC in the United States ^39^. The recent years have brought much progress regarding the genetics of GC and the number of confirmed GC associated SNPs and genes have risen dramatically ^4^ ^,^ ^48^. In addition, several studies have supported the concept that environmental factors are critical components of GC pathogenesis ^52^. However, genetic factors may modify the propensity for GC development through an alteration of the inflammatory state and may also interact with other risk factors ^44^.

Presently the mechanisms of the etiology and progression of GC are far from clear ^4^ ^,^ ^38^. Several genes have been identified to be associated with GC risk, including Interleukin-10 (IL-10). IL-10 is a multifunctional cytokine with anti-inflammatory properties, which has been reported involving in the some malignancies progress and development ^22^ ^,^ ^48^. The human gene that encodes IL-10 (Gene ID: 3586) maps to the long arm of chromosome 1 (1q31-32), which contains three most common 21082A>G (rs1800896), 2592C>A (rs1800872) and 2829C>T (rs1800871) polymorphisms located within the promoter region. These polymorphisms are associated with low/high amount of IL-10 secretion ^31^ ^,^ ^45^.

A series of epidemiological studies have reported the association of -592A>C (rs1800872) and -819T>C (rs3021097) polymorphisms of IL-10 gene with GC risk ^22^ ^,^ ^31^ ^,^ ^45^, but the results remain conflicting rather than conclusive. Some meta-analyses previously published regarding the association of -592A>C (rs1800872) and -819T>C (rs3021097) polymorphisms with GC risk ^4^ ^,^ ^48^ ^,^ ^55^. A few studies were not included in these meta-analyses and also original studies with larger sample sizes in different ethnicity have been published since then. In addition, some of the previous meta-analyses have reported conflicting conclusions. 

Hence, we performed this meta-analysis to evaluate whether the IL-10 -592A>C and -819T>C polymorphisms contributed to the susceptibility of GC. Based on our knowledge, this is the most comprehensive and accurate meta-analysis of the association of IL-10 -592A>C and -819T>C polymorphisms with GC risk.

## METHOD

### Search strategy

The electronic databases of the US National Library of Medicine’s PubMed, EMBASE, Web of Knowledge, Google Scholar, Wanfang, Chinese National Knowledge Infrastructure (CNKI), and Chinese Biomedical Literature Database (CBM) were systematically searched to retrieve potential publications that assessed the association between -592A>C and -819T>C polymorphisms of IL-10 gene and GC risk up to April 10, 2018. Key search terms used were as follows: (gastric cancer OR gastric neoplasm OR stomach neoplasms) AND (Interleukin-10 OR IL-10) AND (-592A>C OR rs1800872) AND (-819T>C OR rs1800871) AND (Polymorphism OR SNP OR single nucleotide polymorphism OR variation OR mutation). This meta-analysis included only publications relating to humans, covering all relevant written in English and Chinese publications with available full-text articles. Reference lists of retrieved articles, review articles, and previous meta-analysis were also manually searched to avoid missing relevant studies.

### Inclusion and exclusion criteria

Studies were included in the meta-analysis if they met the following criteria: 1) full text available; 2) case-control or cohort studies; 3) studies focus on the association of -592A>C (rs1800872) and -819T>C (rs3021097) polymorphisms of IL-10 gene with GC risk; 4) sufficient published data for genotype and allele frequencies to calculate the Odds Ratio (OR) and 95% confidence interval (CI). 

Major reasons for exclusion of studies were as follows: 1) abstract, review articles, case reports, unpublished data and comments; 2) studies with overlapped or duplicate data; 3) no healthy control group established in the study; 4) studies with unclear or ambiguous data or genotype frequencies. When duplicated studies were published by the same author obtained from the same patient sample, only the one with the largest sample size was included in this meta-analysis.

### Data extraction

Data were carefully extracted from all eligible studies independently by two investigators according to the inclusion and exclusion criteria. The following data were collected from each study: first author, year of publication, country origin, ethnicity, total number of cases and controls, the frequencies of genotypes, minor allele frequencies (MAFs), p-value for Hardy-Weinberg equilibrium (HWE). In case of disagreement (in the data extraction), consensus was resolved through consensus, or a third author would assess these articles. In the current meta-analysis, the quality of selected studies was tested by the confirmation of HWE in control groups, and studies without the confirmation of HWE in controls were defined as low-quality studies, while studies with the confirmation of HWE in controls were defined as high-quality studies (Table 1).

**TABLE 1:** The general characteristics of eligible studies in the meta-analysis of IL-10 -592A>C

First Author	Country (Ethnicity)	Case	Control	Cases				Controls				MAFs		HWE	
				Genotypes			Allele		Genotypes			Allele			
				AA	CA	CC	A	C	AA	CA	CC	A	C		
Wu 2003 ^46^	China (Asian)	220	230	88	105	27	281	159	127	83	20	337	123	0.267	0.231
El-Omar 2003 ^10^	USA(Caucasian)	314	210	35	101	178	171	457	13	70	127	96	324	0.771	=0.001
Savage 2004 ^37^	China (Asian)	84	386	9	39	36	57	111	49	166	171	205	567	0.734	0.382
Zambon 2005 ^53^	Italy (Caucasian)	129	644	17	42	70	76	182	46	245	353	337	951	0.738	0.696
Alpizar-Alpizar 2005 ^1^	Costa Rica(Latinos)	45	45	3	20	21	27	63	5	21	18	32	58	0.647	0.761
Lee 2005 ^25^	Korea (Asian)	122	120	52	62	8	166	78	53	60	7	166	74	0.308	0.059
Kamangar 2006 ^19^	Finland (Caucasian)	112	237	6	38	68	50	174	17	82	109	132	342	0.721	0.775
Sicinschi 2006 ^39^	Mexico (Latinos)	181	369	40	90	51	170	192	95	176	98	366	372	0.504	0.376
Sugimoto 2007 ^43^	Japan (Asian)	105	168	43	54	8	140	70	88	70	10	246	90	0.267	0.419
Garcia 2008 ^11^	Spain (Caucasian)	404	404	24	143	237	191	617	28	131	245	187	621	0.768	0.075
Crusius 2008 ^5^	Netherland (Caucasian)	237	1122	11	78	148	100	374	83	397	642	563	1681	0.749	0.049
Deng 2008 ^8^	China (Asian)	125	110	30	39	56	99	151	39	25	46	103	117	0.531	=0.001
Xiao 2009 ^47^	China (Asian)	220	624	100	100	20	300	140	272	283	69	1038	210	0.337	0.718
Kang 2009 ^20^	Korea (Asian)	333	332	142	157	34	441	225	146	145	41	437	227	0.341	0.591
Con 2009 ^3^	Costa Rica(Latinos)	52	191	10	26	16	44	60	23	65	103	111	271	0.709	0.015
Oh 2010 ^33^	China (Asian)	178	362	77	81	20	235	121	167	159	36	493	231	0.319	0.861
Liu 2011 ^27^	China (Asian)	234	243	99	96	39	294	174	109	106	28	324	162	0.333	0.772
He 2012 ^14^	China (Asian)	196	248	82	96	18	260	132	92	128	28	312	184	0.371	0.095
Zeng 2012 ^54^	China (Asian)	151	153	59	77	15	195	107	80	66	7	226	80	0.261	0.147
Kim 2012 ^22^	Korea (Asian)	495	495	231	214	50	676	314	248	191	56	687	303	0.306	0.041
Pan 2013 ^34^	China (Asian)	308	308	144	128	36	416	200	142	135	31	419	197	0.319	0.895
Kuo 2014 ^24^	China (Asian)	358	358	186	134	38	506	210	358	180	141	501	215	0.340	=0.001
Hormazabal 2014 ^31^	Chile (Latinos)	147	172	19	73	55	111	183	11	83	78	105	239	0.694	0.070
Yin 2015 ^50^	China (Asian)	228	461	112	96	20	320	136	235	184	42	654	268	0.290	0.490
de Oliveira 2015 ^6^	Brazil (Latinos)	207	240	104	82	21	290	124	169	64	7	402	78	0.162	0.753
Ma 2016 ^28^	China (Asian)	147	150	67	63	17	197	97	71	67	12	208	92	0.303	0.486

### Statistical analysis

All meta-analyses were conducted using Comprehensive Meta-Analysis (CMA) software (USA, version 2.2.064) and a p value below 0.05 was considered statistically significant. The strength of the association of -592A>C and -819T>C polymorphisms of IL-10 gene with GC risk was estimated by crude odds ratios (ORs) with corresponding 95% confidence intervals (CIs). The significance of the pooled OR was determined by the Z-test. An allele contrast model (C vs. T), homozygote model (CC vs. TT), heterozygote model (CT vs. TT), dominant (CC+CT vs. TT), and recessive (CC vs. CT+TT) model were used for IL-10 -819T>C polymorphism. An allele contrast model (C vs. A), homozygote model (CC vs. AA), heterozygote model (CA vs. AA), dominant (CC+CA vs. AA), and recessive (CC vs. CA+AA) model were used for -592A>C polymorphism. The Cochran chi-square-based Q statistical test was used to evaluate statistical between-study heterogeneity (with p<0.05 for statistical significance). In addition, a quantitative measure of between-study heterogeneity was also investigated using the I^2^ statistic, and which the between-study heterogeneity was considered low, moderate, and high based on I^2^ values of 25%, 50%, and 75%, respectively^18^. If the between-study heterogeneity was statistically significant the random effects model^7^ was applied; otherwise, the fixed effects model ^29^ was used. The sensitivity analysis was performed to assess the contribution of individual studies to pooled effect estimate by sequentially removing each study one at a time and computing differential estimates for rest. In addition, sensitivity analysis was performed by excluding the low quality studies to test the stability of the results. Publication bias was examined using the Begg’s funnel plot and Egger’s test ^2^ ^,^ ^9^. If publication bias existed, the Duval and Tweedie non-parametric ‘’trim and fill’’ method was used to adjust for it. Subgroup analyses by ethnicity and studies quality (by HWE status) were performed subsequently. The distribution of genotypes in control groups was evaluated for a departure from HWE using chi-square test.

## RESULTS

### Characteristics of studies

**FIGURE 1:**
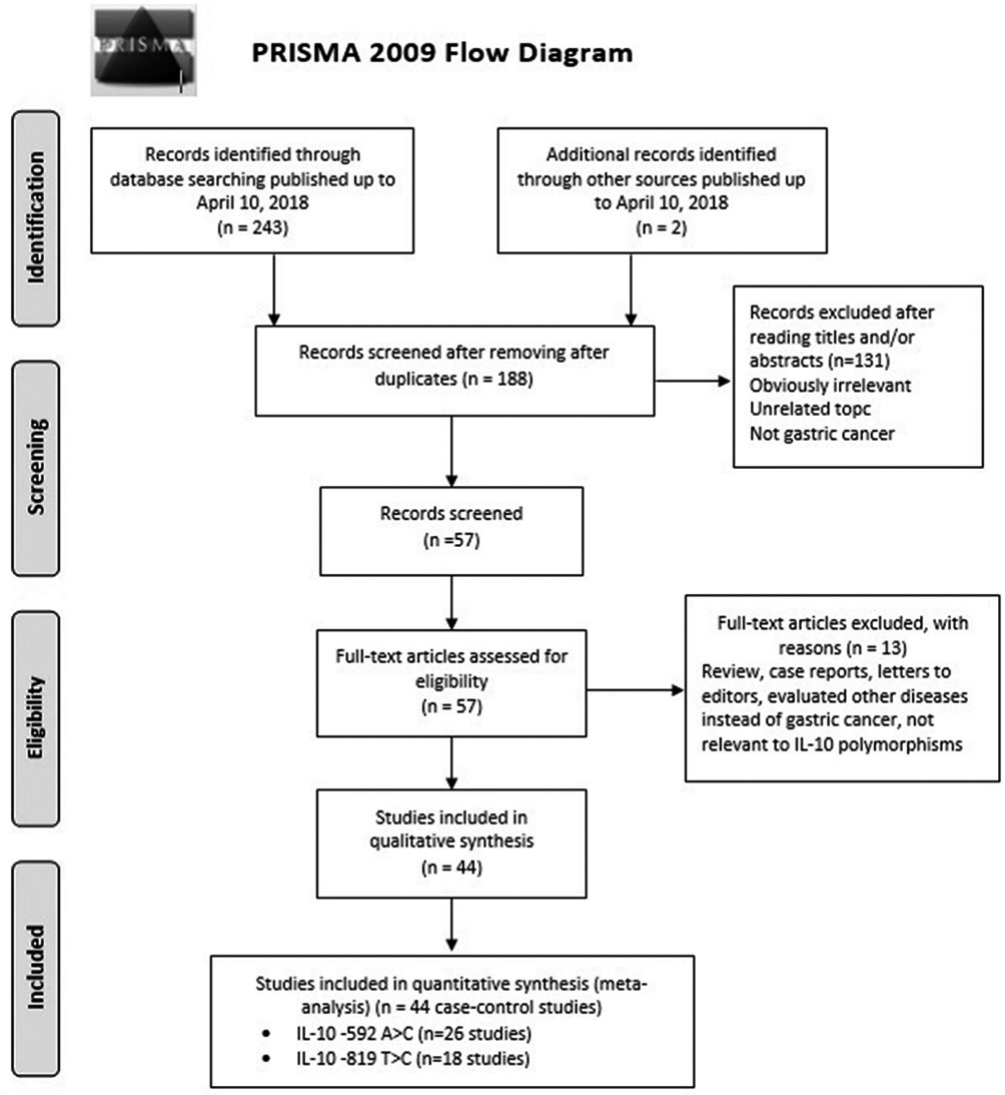
Flow chart of studies selection in this meta-analysis (IL-10 -592A>C and -819T>C Polymorphisms)

Tables 1 and 2 showed the characteristics of all the eligible studies selected in the meta-analysis. The study selection processes were presented in Figure 1 (PRISMA 2009 Flow Diagram). We evaluated all the retrieved studies by examining titles, abstracts and conclusions. According to the criteria eligibility, 44 studies in 29 publications was identified regarding the association between the IL-10 -592A>C and -819T>C polymorphisms with susceptibility to the GC. All of these 44 case-control studies provided sufficient data to calculate the association between the IL-10 -592A>C ^1^ ^,^ ^3^ ^,^ ^5^ ^,^ ^6^ ^,^ ^8^ ^,^ ^10^ ^,^ ^11^ ^,^ ^14^ ^,^ ^19^ ^,^ ^20^ ^,^ ^22^ ^,^ ^24^ ^,^ ^25^ ^,^ ^27^ ^,^ ^28^ ^,^ ^31^ ^,^ ^33^ ^,^ ^34^ ^,^ ^43^ ^,^ ^46^ ^,^ ^47^ ^,^ ^37^ ^,^ ^39^ ^,^ ^50^ ^,^ ^53^ ^,^ ^54^ and -819T>C ^1^ ^,^ ^5^ ^,^ ^14^ ^,^ ^19^ ^,^ ^22^ ^-^ ^24^ ^,^ ^26^ ^,^ ^27^ ^,^ ^33^ ^,^ ^37^ ^,^ ^43^ ^,^ ^42^ ^,^ ^46^ ^,^ ^47^ ^,^ ^51^ ^,^ ^53^ ^,^ ^54^ polymorphisms with risk of GC. The characteristics of the selected studies are summarized in Tables 1 and 3. Among these studies, 26 case-control studies evaluated the association of the -592A>C polymorphism with GC with 5,332 cases and 8,272 controls, included five groups of Caucasians ^5^ ^,^ ^10^ ^,^ ^11^ ^,^ ^19^ ^,^ ^53^, 16 groups of Asians ^8^ ^,^ ^14^ ^,^ ^20^ ^,^ ^22^ ^,^ ^24^ ^,^ ^25^ ^,^ ^27^ ^,^ ^28^ ^,^ ^33^ ^,^ ^34^ ^,^ ^37^ ^,^ ^43^ ^,^ ^47^ ^,^ ^46^ ^,^ ^50^ ^,^ ^54^, and five Latinos populations ^1^ ^,^ ^3^ ^,^ ^6^ ^,^ ^31^ ^,^ ^39^ (Table 1). While, 18 case-control studies evaluated the association between the -819T>C polymorphisms and GC risk, with 3,431 cases and 6,109 controls, included three groups of Caucasians ^5^ ^,^ ^19^ ^,^ ^53^, 14 groups of Asians ^14^ ^,^ ^22^ ^-^ ^24^ ^,^ ^26^ ^,^ ^27^ ^,^ ^33^ ^,^ ^37^ ^,^ ^42^ ^,^ ^43^ ^,^ ^46^ ^,^ ^47^ ^,^ ^51^ ^,^ ^54^, and one Latinos populations^1^ (Table 2). The countries of these studies included China, Korea, Japan, India, USA, Italy, Finland, Spain, Netherland, Costa Rica, Brazil, Mexico and Chile. All the genotype distributions of controls were in agreement with HWE for IL-10 -592A>C and -819T>C polymorphisms except for nine studies in five publications^11^ ^,^ ^21^ ^,^ ^30^ ^,^ ^31^ ^,^ ^40^. Therefore, 35 of 44 case-control studies were defined as high-quality studies (Tables 1 and 2).

**TABLE 2:** The general characteristics of eligible studies in the meta-analysis of IL-10 -819T>C

First Author	Country (Ethnicity)	Case	Control	Cases				Controls				MAFs		HWE	
				Genotypes			Allele		Genotypes			Allele			
				TT	CT	CC	T	C	TT	CT	CC	T	C		
Wu 2003 ^46^	China (Asian)	220	230	88	105	27	281	159	127	83	20	337	123	0.267	0.231
Savage 2004 ^37^	China (Asian)	84	382	37	38	9	112	56	170	163	49	503	261	0.341	0.314
Zambon 2005 ^53^	Italy (Caucasian)	129	644	17	42	70	76	182	46	245	353	337	951	0.738	0.696
Alpizar-Alpizar 2005 ^1^	Costa Rica (Latinos)	45	45	4	16	25	24	66	3	24	18	30	60	0.666	0.179
Kamangar 2006 ^19^	Finland (Caucasian)	98	152	5	35	58	45	151	10	62	80	114	222	0.730	0.662
Sugimoto 2007 ^43^	Japan (Asian)	105	168	42	57	6	141	69	86	73	9	245	91	0.270	0.194
Crusius 2008 ^5^	European (Caucasian)	229	1094	12	72	145	96	362	80	378	636	538	1650	0.754	0.023
Xiao 2009 ^47^	China (Asian)	220	624	100	100	20	300	140	272	283	69	827	421	0.337	0.718
Oh 2010 ^33^	China (Asian)	188	379	81	87	20	249	127	179	158	42	516	242	0.319	0.425
Su 2010 ^42^	China (Asian)	43	100	18	21	4	57	29	51	43	6	145	55	0.275	0.433
Liu 2011 ^27^	China (Asian)	234	243	99	96	39	294	174	109	106	28	324	162	0.333	0.772
He 2012 ^14^	China (Asian)	196	248	82	96	18	260	132	92	128	28	312	184	0.371	0.095
Yuan 2012 ^51^	China (Asian)	279	296	108	129	42	345	213	142	120	34	404	188	0.317	0.265
Zeng 2012 ^54^	China (Asian)	151	153	60	80	11	200	102	78	65	10	221	85	0.277	0.466
Kim 2012 ^22^	Korea (Asian)	495	495	231	214	50	676	314	248	191	56	687	303	0.306	0.041
Kuo 2014 ^24^	China (Asian)	358	358	190	132	36	512	204	186	132	40	504	212	0.296	0.028
Kumar 2015 ^23^	India (Asian)	200	250	36	103	61	175	225	30	119	101	179	321	0.642	0.574
Li 2016 ^26^	China (Asian)	157	248	36	83	38	155	159	36	127	85	199	297	0.598	0.300

### Meta-analysis

#### IL-10 -592A>C Polymorphism

**FIGURE 2:**
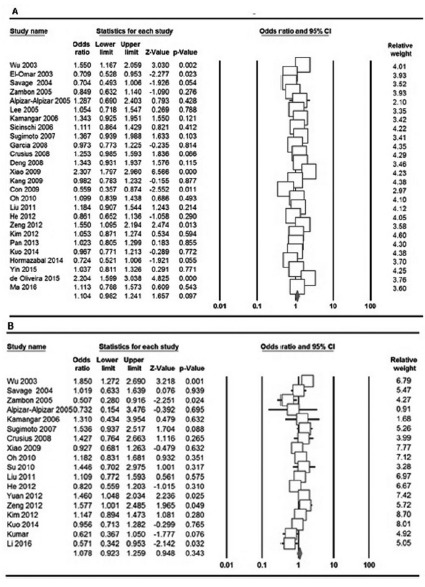
Forest plot of the association of IL-10 -592A>C and -819T>C Polymorphisms with GC: A) -592A>C (homozygote model: C vs. A); B) -819T>C (dominant model: CC+CT vs. TT)

Table 3 listed the main results of the meta-analysis of IL-10 -592A>C polymorphism and GC risk. When all the eligible studies were pooled into the meta-analysis of IL-10 -592A>C polymorphism, a significant association was found only under the heterozygote model (CA vs. AA: OR=1.153, 95% CI=1.020-1.305, p=0.023, Figure 2A). In addition, significant between-study heterogeneity was detected in all genetic models. When stratified by ethnicity, a significant association between of IL-10 -592A>C polymorphism and increased GC risk among Asians was detected under the allele model (C vs. A: OR=1.153, 95% CI=1.007-1.320, p=0.040) and the heterozygote model (CA vs. AA: OR=1.218, 95% CI=1.076-1.379, p= 0.002), but not among Caucasian and Latinos populations. Subgroup analysis of studies with high quality showed that there was a significant association between IL-10 -1082 A>G polymorphism and increased risk of GC only under the allele model (OR=1.154, 95% CI=1.004-1.326, p=0.044, Table 2).

**TABLE 3:** The meta-analysis of IL-10 -592A>C polymorphism and risk of GC

Subgroup	Study number	Genetic model	Type of model	Heterogeneity		Odds ratio				Publication Bias	
				I2 (%)	PH	OR	95% CI	Ztest	POR	PBeggs	PEggers
Overall	26	C vs. A	Random	76.40	=0.001	1.104	0.982-1.241	1.657	0.097	0.724	0.974
	26	CC vs. AA	Random	63.55	=0.001	1.081	0.868-1.345	0.694	0.488	0.427	0.401
	26	CA vs. AA	Random	44.34	0.009	1.153	1.020-1.305	2.268	0.023	0.860	0.569
	26	CC+CA vs. AA	Random	89.63	=0.001	1.085	0.828-1.422	0.589	0.556	0.964	0.559
	26	CC vs. CA+ AA	Random	77.34	=0.001	1.003	0.815-1.235	0.030	0.976	0.171	0.254
By Ethnicity											
Caucasian	5	C vs. A	Random	67.19	0.016	0.992	0.797-1.235	-0.007	0.944	0.806	0.953
	5	CC vs. AA	Random	65.33	0.021	0.959	0.572-1.608	-0.157	0.875	0.806	0.601
	5	CA vs. AA	Random	60.26	0.039	0.891	0.540-1.470	-0.452	0.651	1.000	0.869
	5	CC+CA vs. AA	Random	81.47	=0.001	1.125	0.569-2.223	0.339	0.735	0.462	0.252
	5	CC vs. CA+ AA	Random	55.56	0.061	1.071	0.922-1.245	0.895	0.371	0.462	0.456
Asian	17	C vs. A	Random	73.59	0.001	1.153	1.007-1.320	2.057	0.040	0.224	0.664
	17	CC vs. AA	Random	59.74	0.001	1.193	0.937-1.519	1.429	0.153	0.029	0.003
	17	CA vs. AA	Random	40.21	0.044	1.218	1.076-1.379	3.111	0.002	0.536	0.356
	17	CC+CA vs. AA	Random	92.39	=0.001	1.133	0.810-1.585	0.728	0.467	0.483	0.648
	17	CC vs. CA+ AA	Random	81.80	=0.001	1.050	0.755-1.461	0.290	0.771	0.052	0.013
Latinos	5	C vs. A	Random	87.97	=0.001	1.053	0.660-1.681	0.216	0.829	0.806	0.759
	5	CC vs. AA	Random	80.95	0.001	0.518	0.151-1.776	-1.047	0.295	0.308	0.373
	5	CA vs. AA	Fixed	20.76	0.286	1.001	0.707-1.418	0.007	0.995	1.000	0.737
	5	CC+CA vs. AA	Fixed	55.11	0.083	0.925	0.667-1.283	-0.469	0.639	1.000	0.591
	5	CC vs. CA+ AA	Random	65.75	0.033	0.787	0.491-1.261	-0.997	0.319	0.734	0.757
High Quality Studies											
	20	C vs. A	Random	77.22	=0.001	1.154	1.004-1.326	2.012	0.044	0.417	0.791
	20	CC vs. AA	Random	54.37	0.002	1.191	0.989-1.342	1.820	0.069	0.381	0.717
	20	CA vs. AA	Random	45.14	0.015	1.131	0.982-1.304	1.710	0.087	0.721	0.873
	20	CC+CA vs. AA	Random	63.33	=0.001	1.176	0.997-1.387	1.930	0.054	0.256	0.630
	20	CC vs. CA+ AA	Fixed	31.81	0.086	1.079	0.961-1.211	1.285	0.199	0.040	0.029

#### IL-10 -819T>C Polymorphism

Table 4 and Figure 2B showed the main results of the meta-analysis of IL-10 -819T>C polymorphism and GC risk. When all the eligible studies were pooled into the meta-analysis of IL-10 -819T>C polymorphism, no significant association was observed in any genetic model. In the stratified analyses based on ethnicity and studies quality, there was not still significant association between IL-10 -819T>C polymorphism and risk of GC.

**TABLE 4:** The meta-analysis of IL-10 -819T>C polymorphism and risk of GC

Subgroup	Study number	Genetic model	Type of model	Heterogeneity			Odds ratio			Publication Bias	
				I2 (%)	PH	OR	95% CI	Ztest	POR	PBeggs	PEggers
Overall	18	C vs. T	Random	58.48	0.001	1.057	0.950-1.177	1.017	0.309	0.820	0.381
	18	CC vs. TT	Random	46.47	0.016	0.987	0.795-1.225	-0.120	0.905	0.544	0.469
	18	CT vs. TT	Random	44.86	0.021	1.092	0.943-1.264	1.171	0.242	0.324	0.376
	18	CC+CT vs. TT	Random	55.29	0.002	1.078	0.923-1.259	0.948	0.343	0.404	0.621
	18	CC vs. CT+ TT	Fixed	25.96	0.150	1.003	0.890-1.131	0.056	0.955	0.448	0.492
By Ethnicity											
Caucasian	3	C vs. T	Fixed	50.64	0.132	1.086	0.914-1.289	0.937	0.349	1.000	0.982
	3	CC vs. TT	Random	66.66	0.050	1.008	0.474-2.144	0.021	0.983	1.000	0.753
	3	CT vs. TT	Fixed	59.86	0.083	0.803	0.524-1.232	-1.004	0.315	1.000	0.799
	3	CC+CT vs. TT	Random	67.42	0.046	0.938	0.445-1.980	-0.167	0.867	1.000	0.744
	3	CC vs. CT+ TT	Fixed	0.00	0.552	1.163	0.941-1.438	1.398	0.162	1.000	0.979
Asian	14	C vs. T	Random	63.82	0.001	1.046	0.924-1.184	0.708	0.479	0.742	0.499
	14	CC vs. TT	Random	49.48	0.018	0.987	0.778-1.254	-0.104	0.917	0.661	0.545
	14	CT vs. TT	Random	42.40	0.047	1.132	0.980-1.307	1.684	0.092	0.742	0.879
	14	CC+CT vs. TT	Random	57.22	0.004	1.105	0.942-1.295	1.224	0.221	0.584	0.826
	14	CC vs. CT+ TT	Fixed	20.33	0.232	0.917	0.792-1.062	-1.157	0.247	0.125	0.170
High Quality Studies											
	15	C vs. T	Random	54.22	0.006	1.085	0.966-1.219	1.377	0.169	0.552	0.391
	15	CC vs. TT	Random	52.77	0.009	0.974	0.742-1.278	-0.191	0.848	0.620	0.488
	15	CT vs. TT	Random	52.86	0.008	1.077	0.894-1.297	0.779	0.436	0.276	0.326
	15	CC+CT vs. TT	Random	61.51	0.001	1.063	0.874-1.294	0.611	0.541	0.198	0.460
	15	CC vs. CT+ TT	Fixed	30.61	0.125	0.980	0.848-1.132	-0.275	0.784	0.322	0.150

### Heterogeneity and sensitivity analysis

As shown in Tables 3 and 4, there was a significant between-study heterogeneity for IL-10 -592A>C polymorphism under all genetic models (C vs. A: Ph=0.001; CC vs. AA: Ph=0.001; CA vs. AA: Ph=0.009; CC+CA vs. AA: Ph=0.001; CC vs. CA+ AA: Ph=0.001), and for of IL-10 -819T>C (rs3021097) polymorphism under four genetic models (C vs. A: Ph=0.001; CC vs. TT: Ph=0.0160.001; CT vs. TT: Ph=021; and CC+CT vs. TT: Ph=0.002), except the recessive genetic model (CC vs. CT+ TT: Ph=0.150). We performed sensitivity analysis by omitting one study at a time and calculating the pooled ORs again. However, the results did not show any significant statistical differences when studies were omitted. Therefore, the stability of the study was not influenced by any individual study.

### Publication bias

**FIGURE 3:**
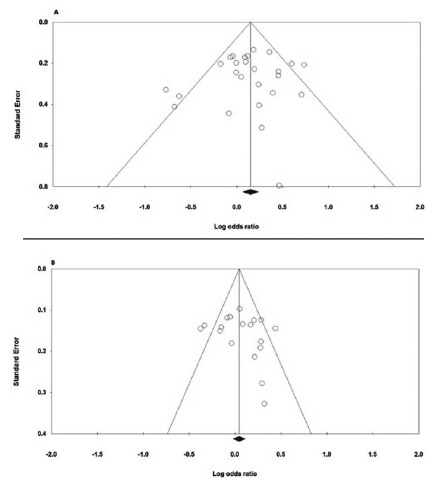
Funnel plot for publication bias in the meta-analysis of the IL-10 -592A>C and -819T>C Polymorphisms with GC: A) -592A>C (heterozygote model: CA vs. AA); B) -819T>C (allele model: C vs. T).

Both Begg’s funnel plot and Egger’s test were carried out to evaluate the publication bias of the studies. Tables 3 and 4 presents the results of Begg’s funnel plot and Egger’s test under the five genetic models. As shown in Figure 3A, the shapes of the Begg’s funnel plots under the allele model of IL-10 -592A>C polymorphism shown approximately symmetrical and significant evidence of publication bias was not observed by the Egger’s test. As for the IL-10 -819T>C polymorphism, the shapes of the Begg’s funnel plots under the heterozygote comparison model seemed symmetrical (Figure 3B). In addition, the Egger’s tests (all p values for Egger’s test>0.05) also showed that there was no evidence of publication bias for both polymorphisms.

## DISCUSSION

A meta-analysis can combine results from individual studies to overcome the limitation of small sample sizes and inadequate statistical power, produce a single estimate of the major effect, answer questions not resolved by the individual studies, resolve controversial debates arising from conflicting studies and cite limitations of current knowledge ^12^ ^,^ ^15^. To date, several meta-analyses have been performed to evaluate the association of the IL-10 gene promoter -592A>C and -819T>C polymorphisms with GC. However, due to lack of ability to obtain overall reliable conclusions because of limited sample sizes, a consensus has not been reached. Therefore, to better elucidate the association of the IL-10 -592A>C and -819T>C polymorphisms with GC, we performed an updated and more comprehensive meta-analysis by collecting 44 relevant case-control studies in 29 publications.

In 2014, Qi et al., in a meta-analysis of twelve studies, with 2,116 GC cases and 4,077 controls, reported that there was no significant association between the IL-10 -592C>A polymorphism and GC risk in overall population ^35^. Since then, a series of better designed case-control studies on the association between IL-10 -592C>A polymorphism and GC were performed. Therefore, their results as a meta-analysis essentially remain an open field. In the current meta-analysis, 26 eligible case-control studies with 5,332 cases and 8,272 controls were identified and analyzed. Our results showed that there was a significant association between the IL-10 -592C>A polymorphism and susceptibility to GC in total population. Moreover, compared with Qi et al meta-analysis the allele genetic model and subgroup analysis among Latinos were also carried out. In this meta-analysis we found that the IL-10 -592C>A polymorphism was associated with GC risk in Asians under the allele model (C vs. A: OR=1.153, 95% CI=1.007-1.320, p=0.040) and the heterozygote model (CA vs. AA: OR=1.218, 95% CI=1.076-1.379, p=0.002). In addition, it is worth noting that the association between IL-10 -592C>A polymorphism and GC risk was significant by studies quality under the allele model (OR=1.154, 95% CI=1.004-1.326, p=0.044).

In 2016, Cui et al., performed a meta-analysis to assess the susceptibility of the IL-10 -819T>C polymorphism to GC including eleven articles with 1,960 cases and 3,705 controls ^4^. Their results suggested that L-10 -819T>C polymorphism has a protective role in susceptibility to GC. Although their results suggested that the IL-10 -819T>C polymorphism might not contribute to the risk of GC; however, these studies were with small number of cases and controls. In the current meta-analysis, we included a total of 18 case-control studies with 3,431 cases and 6,109 controls. The pooled results indicated that there was no obvious association between IL-10 -819T>C polymorphism to GC. Therefore, our meta-analysis not only confirmed Cui et al results, but also provided most reliable statistical results by including more seven case-control studies ^4^.

Heterogeneity between studies is common in the meta-analysis of genetic association studies ^41^ ^,^ ^49^. In each case, the heterogeneity could be a result of different covariates such as ethnicity, sources of controls, sample size, HWE and methods used and so on ^16^ ^,^ ^17^ ^,^ ^30^. In the current meta-analysis, significant between-study heterogeneity was detected across studies under all genetic models and thus we selected the random-effects model to summarize the ORs. Therefore, we performed meta-regression analysis to find the source of between-study heterogeneity. The results showed that ethnicity and studies quality did not contribute to substantial between-study heterogeneity in the current meta-analysis. Moreover, we have performed sensitivity analysis according to sample size and leave-one-out analysis to determine whether modification of the inclusion criteria by removing one study each time affected the results. However, for both IL-10 -592A>C and -819T>C polymorphisms, the sensitivity analyses did not materially affected the original results.

The present meta-analysis has some advantages compared to the previous meta-analyses. However, it does have some limitations that should be taken into account. First, we have included only studies published in the English and Chinese language in this meta-analysis; therefore, publication bias may have occurred. Second, in this meta-analysis the great proportion of statistical power was contributed by the Asian ethnicity. There were not enough studies in Caucasians and Latinos, which limited the statistical power. Moreover, African was one of the three largest ethnics, but we have not found any study on Africans. Third, the current meta-analysis was performed to analyze these polymorphisms separately; however, a haplotype analysis may have been more powerful for finding significant associations with GC. Forth, the ORs extracted from each eligible study were based on unadjusted estimates, while a more precise analysis should be performed in all individual data available, which would allow for the adjustment by other co-variants including age, environmental exposures, smoking status, and other lifestyle factors. Finally, gene-gene and gene-environment interactions which may modulate the GC susceptibility were not addressed in this meta-analysis for the lack of sufficient data.

## CONCLUSION

The current meta-analysis results inconsistent with the previous meta-analyses showed that the IL-10 -592A>C polymorphism contributed to the susceptibility of GC in overall population, particularly in Asian populations. However, the IL-10 -819T>C polymorphism was not associated with an increased risk of GC. Further large well-designed studies are still needed to determine the effects of the IL-10 -592A>C and -819T>C polymorphisms on GC.
